# Dendritic Morphology of Caudal Periaqueductal Gray Projecting Retinal Ganglion Cells in Mongolian Gerbil (*Meriones unguiculatus)*

**DOI:** 10.1371/journal.pone.0103306

**Published:** 2014-07-23

**Authors:** Chaoran Ren, Mingliang Pu, Qi Cui, Kwok-Fai So

**Affiliations:** 1 Guangdong-Hongkong-Macau Institute of CNS Regeneration, Jinan University, Guangzhou, PR China; 2 Guangdong Medical Key Laboratory of Brain Function and Diseases, Jinan University, Guangzhou, PR China; 3 GHM Collaboration and Innovation Center for Tissue Regeneration and Repair, Jinan University, Guangzhou, PR China; 4 Department of Ophthalmology and State Key Laboratory of Brain and Cognitive Sciences, The University of Hong Kong, Hong Kong, PR China; 5 Department of Anatomy, School of Basic Medical Sciences, Peking University, Beijing, PR China; 6 Key Laboratory on Machine Perception (Ministry of Education), Peking University, Beijing, PR China; 7 Key Laboratory for Visual Impairment and Restoration (Ministry of Education), Peking University, Beijing, PR China; Virginia Tech Carilion Research Institute, United States of America

## Abstract

In this study we investigated the morphological features of the caudal periaqueductal gray (cPAG)-projecting retinal ganglion cells (RGCs) in Mongolian gerbils using retrograde labeling, in vitro intracellular injection, confocal microscopy and three-dimensional reconstruction approaches. cPAG-projecting RGCs exhibit small somata (10–17 µm) and irregular dendritic fields (201–298 µm). Sizes of somata and dendritic fields do not show obvious variation at different distance from the optic disk (eccentricity). Dendrites are moderately branched. Morphological analysis (n = 23) reveals that cPAG-projecting RGCs ramified in sublamina ***a*** and ***b*** in the inner plexiform layer. These cells exhibit different stratification patterns based on the thickness of dendritic bands in sublaminas ***a*** and ***b***: majority of analyzed cells (16 out of 23) have two bands of arborizations share similar thickness. The rest of analyzed cells (7 out of 23) exhibit thinner band in sublamina ***a*** than in sublamina ***b***. Together, the present study suggests that cPAG of Mongolian gerbil could receive direct retinal inputs from two types of bistratified RGCs. Furthermore, a small subset of melanopsin-expressing RGCs (total 41 in 6 animals) is shown to innervate the rostral PAG (rPAG). Functional characteristics of these non-visual center projecting RGCs remain to be determined.

## Introduction

Growing evidence has shown a pivotal role of light stimulation in the regulation of non-image-forming (NIF) functions, such as circadian rhythms, conditioned fear and affective behavior [1]–[3]. Unveiling the morphological and physiological features of NIF system is crucial for us to understand the pathogenesis of affective disorders, including anxiety and depression. In our previous work, morphological and physiological properties of direct dorsal raphe nuclei (DRN)-projecting retinal ganglion cells (RGCs) in Mongolian gerbil were described [4]. We also found that the changes in the activities of DRN-projecting Y/alpha cells could directly modulate the level of serotonin in serotonergic neurons in DRN and influence the depressive behavior [5].

Similar to DRN, periaqueductal gray (PAG) in the midbrain is a multifunctional non-visual center. It receives afferents from a wide range of areas in the brain including several mood related centers such as amygdala and prefrontal cortex, and participates in the regulation of a series of important functions, such as conditioned fear, pain and analgesia [6]–[9]. There is also evidence that the caudal PAG (cPAG) receives direct retinal innervations [10], [11]. Recently, a work carried out in genetically modified mouse shows that sparse axonal terminations of melanopsin-expressing RGCs (mRGCs) are present in the rostral PAG (rPAG) [12]. This result suggests that light information transmitted by those PAG-projecting RGCs may directly influence the activity of neurons in PAG. However, dendritic morphology of PAG-projecting RGCs and their topographic organization remain to be determined.

ON-OFF RGCs have been documented across species [13]–[23]. Seven types of bistratified RGCs have been identified in rabbit, which might be correspondent to four to six morphological types of bistratified RGCs in mouse [23]. Although it is known that a subgroup of bistratified RGCs could respond selectively to the direction of motion, functional role of other types remains obscure [24], [25]. Using a highly sensitive neural tracer Cholera toxin B subunit (CTB) retrograde labeling and direct intracellular dye injection techniques in the present study, we identified a subset of RGCs with morphological properties of bistratified RGCs. We found that these RGCs innervated cPAG but not rPAG in Mongolian gerbils, and there was no detectable level of melanopsin in these cells. We also confirm that mRGCs, with M1-like morphology, only send their axon terminals to rPAG. Since PAG is a non-visual center, it is plausible that both conventional and intrinsically light sensitive RGCs participate in the process of NIF light information.

## Materials and Methods

### Animals

Young adult male Mongolian gerbils (Meriones unguiculatus) (65–80 g) were used in this study. Animals were housed in a 12-hour light/12-hour dark cycle and food and water were provided *ad libitum*. All experiments were performed in accordance with the Association for Research in Vision and Ophthalmology (ARVO) Statement for the Use of Animals in Ophthalmic and Vision Research, and approved by competent ethics committee at Jinan University.

### Retrograde labeling

Animals were intraperitoneally anesthetized with a mixture of ketamine hydrochloride (120 mg/kg) and xylazine (15 mg/kg), and placed in a stereotaxic instrument after the confirmation of achieving sufficient anesthesia, in which animals have regular respiratory rate and no corneal reflexes and toe pinch. Animals were kept warm by a heat lamp throughout the experiment. Targeting of the caudal periaqueductal gray (cPAG) was conducted as follows: to avoid the dye diffusion into superior colliculus, a small hole was made using a dental drill (2.6 mm posterior to lambda; 0.8 mm to midline) and a 10 µl Hamilton syringe was inclined at 30° posteriorly from vertical axis and advanced slowly for 5.2 mm. Totally 0.2 µl of Cholera toxin B subunit (CTB)-conjugated Alexa Fluor 488 or 594 (C34777 or C22842, Invitrogen Inc., Grand Island, NY) was slowly injected in 3 minutes. The syringe was held in place for 10 minutes, and then withdrew slowly. Procedures of targeting the rostral PAG was similar to the caudal work except that the small hole was made 2.6 mm posterior to bregma and 0.3 mm to midline, and the syringe was advanced vertically for 4.2 mm. Following CTB injection, the wound was sutured and antibiotics (bacitracin and neomycin) were applied to the surgical wound. To relieve the pain of animals, ketoprofen (5 mg/kg) was injected subcutaneously. Then, animals were allowed to recover from anesthesia under a heat lamp.

### Intracellular injection

The intracellular injection of RGCs that had been retrogradely labeled with CTB was performed as previously described [4], [5]. Briefly, animals were anesthetized (120 mg/kg ketamine and 15 mg/kg xylazine, intraperitoneally), and eyes were enucleated under dim red light. Lens and vitreous were carefully removed with a pair of fine-forceps. The eyecups were flat-mounted, sclera side down, directly on the bottom of a recording chamber and superfused by oxygenated (95% O_2_/5% CO_2_) Ames medium (Sigma-Aldrich, St. Louis, MO) at a fixed rate (5 ml/min) at room temperature (22–24°C). CTB-labeled RGCs were illuminated under epifluorescence microscopy. Once a CTB-labeled cell was identified, an injection electrode containing dyes (4% Lucifer yellow and 3% neurobiotin) was directed towards the cell and penetrated mechanically under visual control. A small biphasic current pulse (up to 2 nA hyperpolarizing and 0.5 nA depolarizing for 1–4 minutes) was applied to inject the dye.

### Immunocytochemistry

Detection of melanopsin-expressing RGCs (mRGCs) was performed as previously described [4], [5]. Briefly, three days after the retrograde labeling, animals were anesthetized and perfused transcardially with 0.9% saline followed by 4% paraformaldehyde in 0.1 M phosphate-buffered saline (PBS). Brains and eyes were removed. Retinas were separated and washed in 0.1 M PBS for 3 times (10 minutes/time) before incubation in 0.1 M PBS containing 10% normal goat serum (Vector Laboratories, Burlingame, CA) and 0.3% Trition-X-100 (T8787, Sigma-Aldrich, St Louis, MO) for 1 hour. Then retinas were incubated for 3 days at 4°C with a rabbit anti-melanopsin antibody (PA1-780, Fisher Scientific Inc., Barrington, IL; 1∶600). This was followed by 6 times rinse in 0.1 M PBS and then incubation with a secondary (Dylight 488 or Dylight 549) goat-anti-rabbit IgG (1∶400, Vector Laboratories) for 6 hours at room temperature.

The method for converting intracellularly injected Lucifer Yellow (Sigma-Aldrich) into permanent dye has been described previously [5]. Briefly, after the injection, retinas were fixed for 1 hour in 4% paraformaldehyde in 0.1 M PBS at room temperature. Retinas were rinsed in 0.1 M PBS for 3 times (10 minutes/time) and placed in 10% normal goat serum containing 2% Trition-X-100 and 0.5% dimethylsulfoxide (Sigma-Aldrich) for 48 hours at 4°C. Retinas were then incubated in rabbit-anti-Lucifer Yellow antibody (1∶1000, Sigma-Aldrich) for 48 hours at 4°C. This was followed by 3 times rinse in 0.1 M PBS and then incubation with a secondary antibody Alexa Fluor 488 goat-anti-rabbit IgG (1∶400, A-21206, Molecular Probes) for 6 hours at room temperature. Finally, all retinas were rinsed in 0.1 M PBS and cover-slipped in anti-fading aqueous mounting medium (EMS, Hatfield, PA).

The method for immunocytochemical staining of serotonergic neurons in dorsal raphe nuclei (DRN) was performed as previously described [5]. Briefly, 40 µm thick cryostat sections collected through the injection site were placed in 0.1 M PBS containing 10% normal goat serum with 0.3% Trition-X-100 for 1 hour before incubation in rabbit anti-serotonin antibody (1∶500, S5545, Sigma-Aldrich) for 72 h at 4°C. Brain sections were then placed in secondary antibody Dylight488 goat-anti-rabbit IgG (1∶200, Vector Laboratories) for 6 hours at room temperature. Finally all sections were rinsed in 0.1 M PBS and cover-slipped in anti-fading aqueous mounting medium (EMS, Hatfield, PA).

The method for immunocytochemical staining of cholinergic amacrine cells in the retina was performed according to the following protocol: retinas were fixed for 1 hour in 4% paraformaldehyde in 0.1 M PBS at room temperature. Retinas were rinsed in 0.1 M PBS for 3 times (10 minutes/time) and placed in 10% normal goat serum containing 2% Trition-X-100 for one hour at room temperature. Retinas were then incubated in goat-anti-ChAT antibody (1∶200, AB144P, Millipore) for 48 hours at 4°C. This was followed by 6 times rinse in 0.1 M PBS and then incubation with a secondary antibody Alexa Fluor 594 donkey-anti-goat IgG (1∶400, A-11058, Molecular Probes) for 6 hours at room temperature. Finally, all retinas were rinsed in 0.1 M PBS and cover-slipped in anti-fading aqueous mounting medium (EMS, Hatfield, PA).

### Confocal microscopy and three-dimensional reconstruction

Immunocytochemically stained RGCs that had been injected with Lucifer Yellow were scanned with a confocal microscope (LSM 700, Carl Zeiss, Germany). The Z-axis interval was 0.3 µm. Each stack of optical sections covered a retinal area of 325.75×325.75 mm^2^ (1024×1024 pixels). Using Image J and Photoshop CS5 (Adobe Corp., San Jose, California, USA), each stack of optical sections were montaged and projected to a 0° X-Y plane and a 90° Y-Z plane to obtain a three-dimensional reconstruction of the cell. Details of three-dimensional reconstruction and confocal calibration procedures were described elsewhere (Pu, 1999). Contrast and brightness were adjusted and the red-green images had been converted to magenta-green.

### Injection site verification

After transcardial perfusion with 0.9% saline followed by 4% paraformaldehyde in 0.1 M PBS, the brain was removed and postfixed with 4% paraformaldehyde overnight at 4°C, and then transferred into 30% sucrose until sectioning with a cryostat (CM1900, Leica Microsystems, Bannockburn, IL). A series of 40 µm brain sections were cut for the verification of injection site and evaluation of whether injections had invaded other visual related nuclei under epifluorescence microscope (Leica, DM6000B).

### Statistical Analysis

Comparison of caliber size of cPAG-projecting RGCs with alpha cells and mRGCs, and density distribution variation within the retina were analysized using the one-way ANOVA test. Data are expressed as the mean ± SEM. Statistical significance was set at p<0.05.

## Results

**Figure 1:**
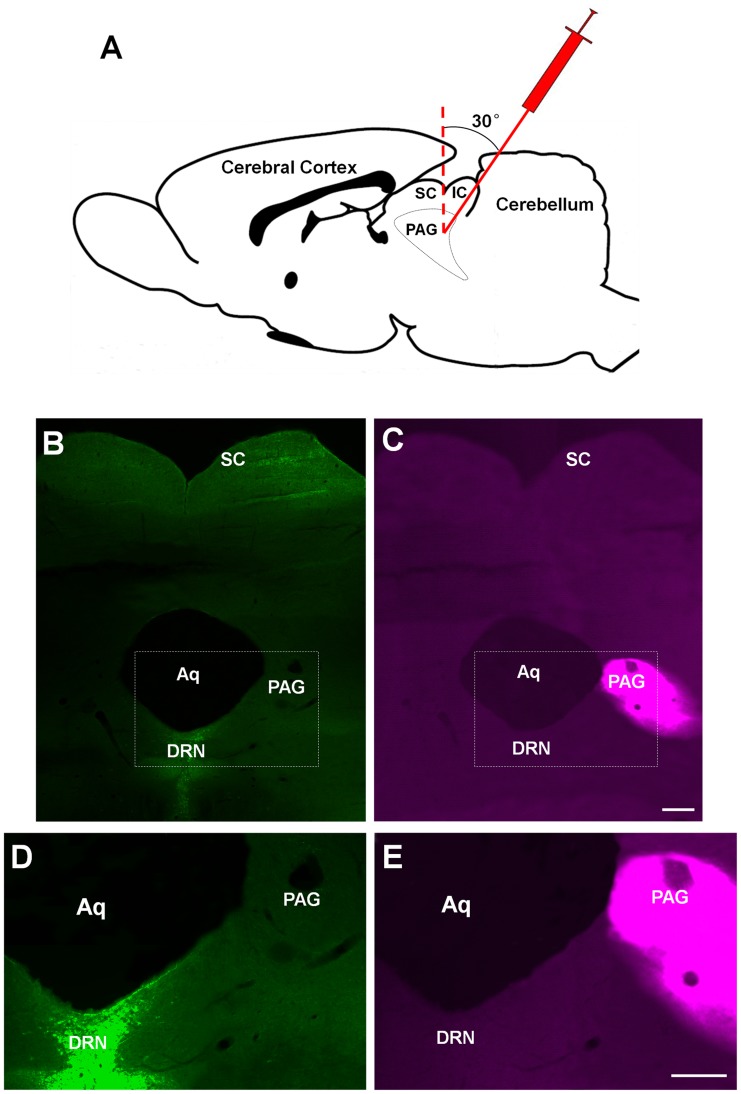
Illustration of the caudal periaqueductal gray (cPAG) injection site. (A) The schematic drawing of Cholera toxin B subunit (CTB) injection. The gray dotted line outlines the border of PAG. Note that to avoid the dye diffusion into superior colliculus (SC), the Hamilton syringe was inclined posteriorly at 30° angle from vertical and advanced 5.2 mm to target the cPAG. (B–E) A coronal section of the injection site. Trace of injection route is shown in (C) and (E). It is evident that CTB did not diffuse into the superior colliculus (SC) and dorsal raphe nuclei (DRN) (C). Large number of 5-HT+ neurons were seen in DRN (green cells in B and D). SC: superior colliculus; IC: inferior colliculus; PAG: periaqueductal gray; DRN: dorsal raphe nuclei; Aq: aqueduct. Scale bars: 300 µm in C (applies to B); 300 µm in E (applies to D).

**Figure 2:**
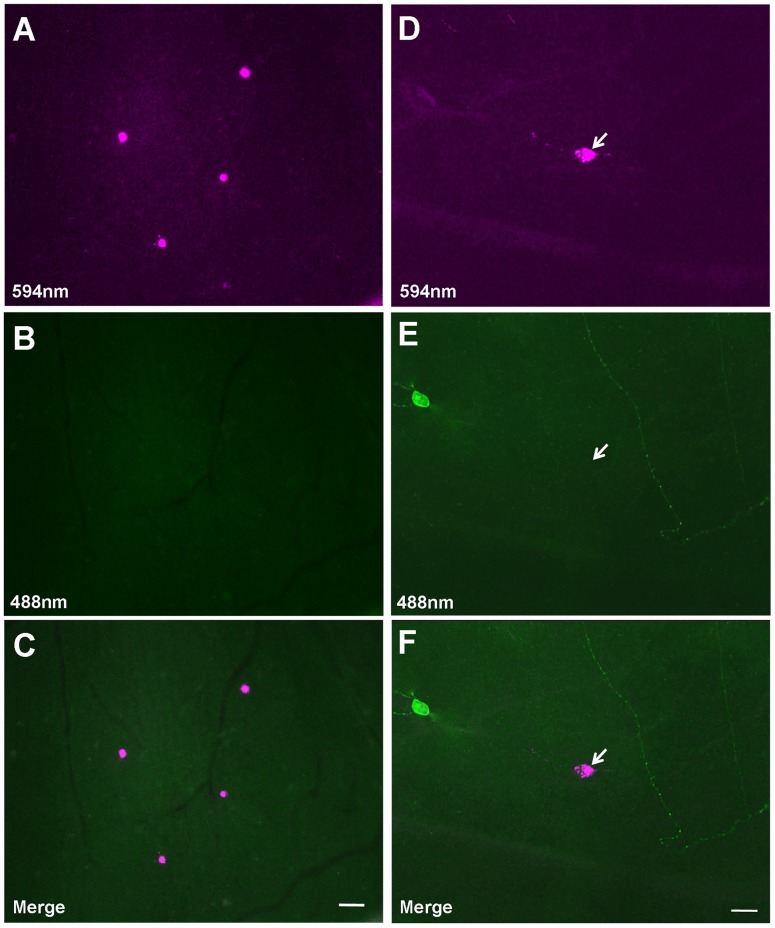
Illustration of Cholera toxin B subunit (CTB) retrogradely labeled-caudal periaqueductal gray (cPAG)-projecting retinal ganglion cells (RGCs). (A–C) Illustration of the CTB-conjugated Alexa Fluor 594 (CTB-594) retrogradely labeled RGCs in the retina. There are 4 CTB-labeled RGCs (magenta) in this field (A). These cells were not visible under blue excitation florescence filter (B). Merged photomicrograph of (A) and (B) is shown in (C). (D–F) A cPAG-projecting RGC is negative for anti-melanopsin staining. Arrow points to a CTB-594 retrogradely labeled cPAG-projecting RGC (D) that, as shown in (E), lacks melanopsin immunoreactivity. In contrast, melanopsin-positive RGCs were also seen. As shown in (E), the soma and dendrites of a melanopsin-expressing RGC (mRGC) were visible, so was an dendrite of another mRGC whose soma was not in the field (green fluorescent staining). Merged photomicrograph shows a cPAG-projecting RGC and a non-cPAG-projecting but melanopsin-positive RGC in the same field (F). Scale bars: 40 µm in C (applies to A and B); 20 µm in F (applies to D and E).

The microinjection setup and retrograde dye deposit location are shown in Fig. 1. To avoid the dye diffusion into the superior colliculus (SC), the injection Hamilton syringe was tilted at 30° backward and reached periaqueductal gray (PAG) through inferior colliculus bypassing the SC (Fig. 1A). By controlling the depth of penetration we also avoided diffusion of the dye into dorsal raphe nuclei (DRN) (Fig. 1B–E), which receives direct afferents from retinal ganglion cells (RGCs) in Mongolian gerbil [4], [11]. Only one injection was made for each animal. Each injection site was verified by examining histological sections, and samples were discarded unless we confirmed that there was no dye diffusion into the SC and DRN. CTB retrogradely labeled caudal PAG (cPAG)-projecting RGCs were selected for intracellular injection (Fig. 2A and 2D). One hundred thirty four cPAG-projecting RGCs that had been retrogradely labeled with CTB in 7 animals were successfully injected, and their morphological characteristics were quantitatively analyzed. Since lines of evidence suggest that melanopsin-expressing RGCs (mRGCs) project to a broad range of central targets including PAG [12], we also examined melanopsin protein in these cPAG-projecting RGCs. However, there were no detectable levels of melanopsin in these cells (Fig. 2D–F).

**Figure 3:**
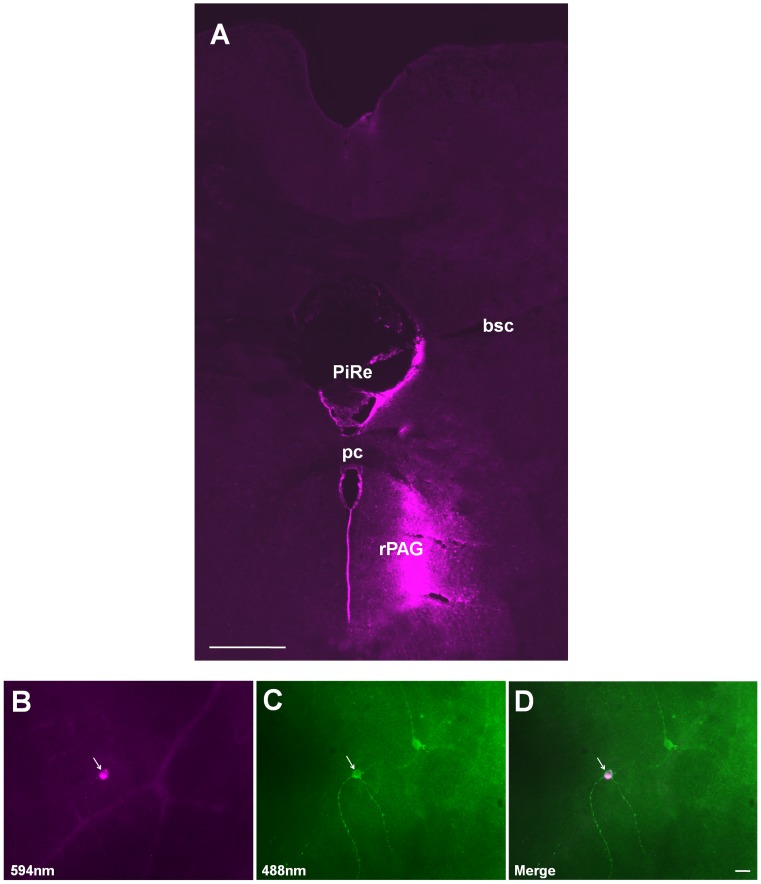
Innervation of melanopsin-expressing retinal ganglion cells (mRGCs) in the rostral periaqueductal gray (rPAG). (A) Coronal section of an injection site. (B–D) Arrow points to a CTB retrogradely labeled rPAG-projecting RGC. This cell was also positive for melanopsin immunoreactivity (C), but not all mRGCs could be retrogradely labeled by CTB (arrowhead in C and D). bsc: the brachium of the superior colliculus; PiRe: pineal recess; pc: posterior commissure; rPAG: the rostral periaqueductal gray. Scale bars: 300 µm in A; 20 µm in D (applies to B and C).

To determine morphology of the rostral PAG (rPAG)-innervating RGCs, CTB tracer was deposited at rPAG (Fig. 3A). Images obtained before and after the immunohistochemical staining for melanopsin protein, and the merged image are shown in Fig. 3B, 3C and 3D, respectively. Of 6 animals with successful rPAG tracer injection, retrogradely labeled RGCs were all melanopsin positive (n = 42), and no conventional (melanopsin negative) RGCs were observed. Our results agree with a previous report that rPAG receives inputs from axon terminals of mRGCs [12].

### Somatic and axonal morphology

**Figure 4:**
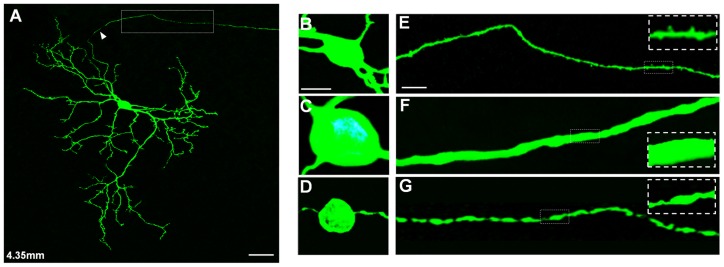
Details in morphology of caudal periaqueductal gray (cPAG)-projecting retinal ganglion cells (RGCs). (A) A photomontage of cPAG-projecting RGC. This cell lies at 4.35 mm from the optic disk. The arrowhead points to an axon. (B–D) High power photomicrographs comparing somata among cPAG-projecting RGC (B), alpha cell (C) and melanopsin-expressing RGC (D). (E–G) High power photomicrographs comparing axons among cPAG-projecting RGC (E), alpha cell (F) and melanopsin-expressing RGC (G). The white frames highlight parts of their axons, respetively. Scale bars: 20 µm in A; 10 µm in B (applies to C and D); 5 µm in E (applies to F and G).

**Figure 5:**
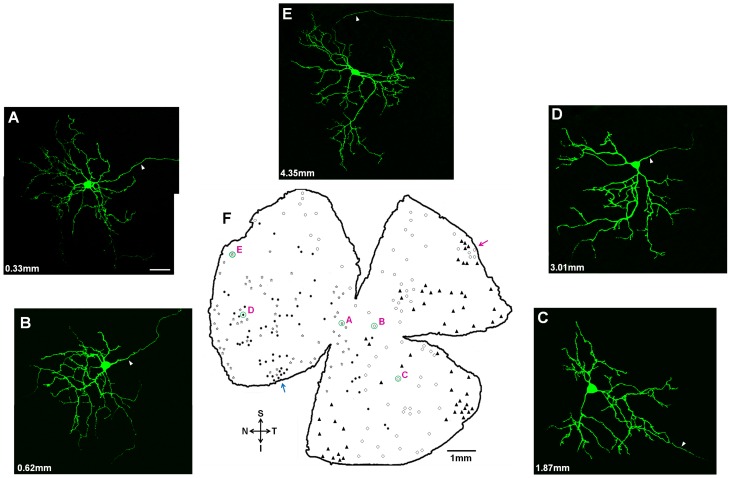
Dendritic morphology and distribution pattern of caudal periaqueductal gray (cPAG)-projecting retinal ganglion cells (RGCs). (A–E) Morphology of intracellularly injected cPAG-projecting RGCs located at different parts of the retina. Arrowheads point to axons. The distances from these cells to the optic disk are: 0.33 mm in (A), 0.62 mm in (B), 1.87 mm in (C), 3.01 mm in (D) and 4.35 mm in (E). (F) Schematic representation of the distribution pattern of Cholera toxin B subunit (CTB) retrogradely labeled cPAG-projecting RGCs from 5 animals. Coordinates of needle tips in 5 animals: •: Lambda: −3.2 mm, midline: left 1.05 mm, depth: 2.73 mm; ☆: Lambda: −3.208 mm, midline: left 0.92 mm, depth: 2.69 mm; ▴: Lambda: −3.204 mm, midline: left 1.08 mm, depth: 2.72 mm; ◊: Lambda: −3.212 mm, midline: left 1.08 mm, depth: 2.64 mm; ○: Lambda: −3.192 mm, midline: left 1.05 mm, depth: 2.82 mm; Green circled cells of A–E are correspondent to the Figure 5A–5E, respectively. One peripheral area (magenta arrow) and two central areas (blue arrow and cell D) have similar local densities. S: superior; I: inferior; N: nasal; T: temporal. Scale bars: 20 µm in A (applies to B–E); 1 mm in F.

As shown in Fig. 4 and 5, cPAG-projecting RGCs have round or ovoid soma, with diameter ranging from 9–17 µm (mean ± S.D., 14.2±3.4 µm; n = 134) (Fig. 4B). Generally, the soma size changed little with retinal eccentricity. The soma sizes in the central 1 mm varied from 11 to 14 µm (n = 9), and the rest of cPAG-projecting cells had relatively large variation (9–17 µm, n = 125) and slightly larger than those in the central retina. Their somata were smaller than alpha cells that were stained with the same method in the same species (22±8 µm; [4]) (Fig. 4C). The cell bodies of cPAG-projecting RGCs were larger than that of dorsal lateral geniculate nucleus (dLGN) innervating cells (11.7±0.06 µm) but similar to the average soma size of RGCs in the gerbil retina (13.4±0.04 µm) [11].

Well-stained axons were slim and curved (Fig. 4A and 4E). The average axon caliber size of cPAG-projecting RGCs was about 0.45±0.12 µm (measured at 50 µm from the center of each soma; n = 95), which is significantly slimmer than alpha cells (2.71±0.36 µm; n = 21; p<0.0001; [4]) (Fig. 4F) and mRGCs (1.03±0.27 µm; n = 86; p<0.001) in the same species (Fig. 4G). As shown in the insert of Fig. 4E, an interesting morphological property of cPAG-projecting RGCs is that their axons bore many spines (Fig. 4E).

### Dendritic morphology

**Figure 6:**
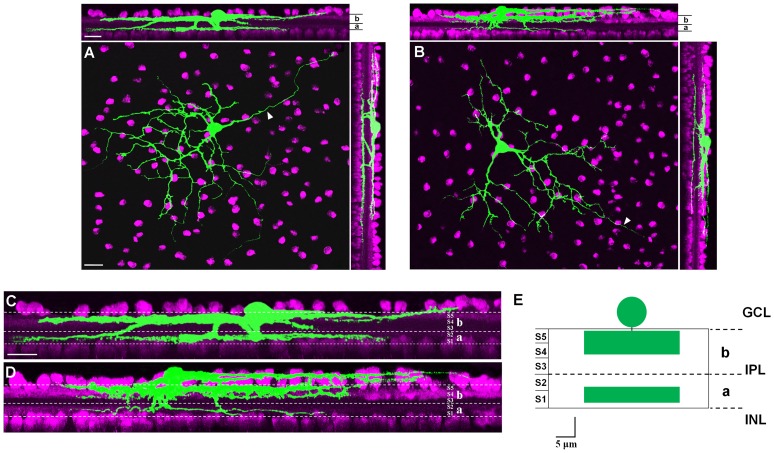
Dendritic stratification of caudal periaqueductal gray (cPAG)-projecting retinal ganglion cells (RGCs). (A–B) These two cells are distinctly bistratified in sublamina ***a*** and ***b*** of the inner plexiform layer (IPL). Cholinergic amacrine cells were labeled with color magenta. (C–D) High-power images of upper planes in (A) and (B), illustrating that these two cPAG-projecting RGCs could distinctly bistratified in IPL. White dotted lines indicated borders of sublamina ***a*** and ***b*** of IPL. (E) Schematic summary of ramification pattern of cPAG-projecting RGCs. INL: inner nuclei layer; IPL: inner plexiform layer; GCL: retinal ganglion cell layer; a: sublamina ***a*** of inner plexiform layer; b: sublamina ***b*** of inner plexiform layer; S1–S5: stratum 1–5. Scale bars: 20 µm in A (applies to B); 20 µm in upper panel of A (applies to upper panel of B); 20 µm in C (applies to D); 5 µm in E.

Most cells gave rise to 2–7 primary dendrites that were generally thick and smooth but varied in length. The primary dendrites branched for 2–8 times. The secondary processes of many cells travelled for long distance (Fig. 5C–E), and some others branched immediately into high order curly processes (Fig. 5A–B). Intermediate branches had irregularly oriented processes and some dendrites coursed centrifugally from the soma. Each cell usually had one or two long terminal processes that frequently consisted of a couple of short processes or thorns. Note that curved dendrites in the periphery field sometimes crossed with each other. The caliber size of dendritic processes also varied in the periphery field. Processes ramified in the sublamina ***a*** usually had slimmer caliber processes whereas those ramified in the sublamina ***b*** usually had thicker ones (Fig. 6A–B). Together, the dendritic field was covered by loosely distributed and occasionally overlapped processes that had many short thorns in their pre-terminal or terminal processes. In comparison with the field size of cells sampled from different locations of the retina, as shown in Fig. 5, the average field size did not vary significantly with eccentricity. On the other hand, the locations of somata among dendritic fields were variable, 42% of them (n = 56) were centered within the field (Fig. 5A) whereas another 58% (n = 78) were eccentric (Fig. 5B–D).

### Ramification pattern

Dendritic field ramification pattern of 23 cPAG-projecting RGCs was quantitatively analyzed. All of them had bistratified dendritic process ramification in sublamina ***a*** and ***b*** in the inner plexiform layer (IPL). However, these bistratified cells showed a slightly different patterns of ramification in sublaminas ***a*** and ***b***. As shown in Fig. 6A and 6C, this cell exhibited two distinctive narrow bands of arborizations in sublaminas ***a*** and ***b*** *.* The two bands of stratification shared similar thickness. The majority of analyzed cells showed this type of ramification pattern (16 out of 23). On the other hand, the rest of analyzed cells (7 out of 23) had a different pattern of stratification. As shown in Fig. 6b and 6D, the sublamina ***b*** was formed by processes arborized at different levels of IPL, but sublamina ***a*** was only occupied by a couple of dendritic processes. Nevertheless, these cells shared a common bistratified ramification pattern. Thus, we quantitatively analyzed ramification patterns of these cells together. As shown in Fig. 6E, the average outer arbors of these cells centered at S4/5 (3.83±3.01 µm), and the inner tiers were confined to S1/2 (16.84±2.07 µm). The average thickness of stratification in sublaminas ***a*** and ***b*** were 3.12±0.76 µm and 7.03±0.51 µm, respectively.

**Figure 7:**
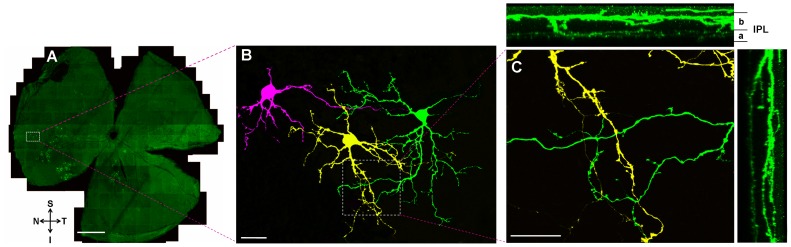
Dendritic fields that sometimes overlap with one another among caudal periaqueductal gray (cPAG)-projecting retinal ganglion cells (RGCs). (A) Intracellularly injected cPAG-projecting RGCs in a whole-mounted retina. S: superior; I: inferior; N: nasal; T: temporal. (B) The white box in (A) viewed under high magnification. In this field three neighboring cPAG-projecting RGCs that have overlapped dendritic fields were labeled with three different colors (magenta, yellow and green). (C) Three dimensional reconstruction of overlapped dendritic fields. IPL: inner plexiform layer; a: sublamina *a* of inner plexiform layer; b: sublamina *b* of inner plexiform layer. Scale bars: 1 mm in A; 20 µm in B; 10 µm in C.

Fig. 7A shows 3 intracellularly injected cPAG-projecting RGCs, Ramification pattern of 2 of them are analysized, and they are bistratified cells (labeled with color yellow and green) (Fig. 7B and C). Fig. 7C depicts small calibered processes stratified at sublamina ***a*** whereas thick ones occupy sublamina ***b***. As shown in the Fig. 7B, dendrites of the two cells overlap with each other. Three-dimensional reconstruction of the overlapping area shows that these two cells co-stratify within the same sublamina in the IPL (Fig. 7C).

### Distribution pattern and coverage factor

By accurately placing the Hamilton syringe and precisely adjusting the injection needle tip depth, we deposited the CTB tracer at the right side of cPAG and retrogradely labeled cPAG-projecting RGCs at the contra-lateral retina. To improve accuracy, only one precision tracer injection was performed for each animal. Five animals with precise tracer deposits at the cPAG were used in this study, and the cPAG-projecting RGC distribution patterns from these retinas were collected and quantitatively analyzed. Fig. 5F shows the summarized distribution pattern of cPAG-projecting RGCs. The CTB tracer deposition (−3.20 mm AP, 1.05 mm ML and 2.73 mm DV) in one animal retrogradely labeled cPAG-projecting RGCs throughout the nasal retina. The other four injections labeled cells in inferior nasal (−3.21 mm AP, 0.92 mm ML, 2.69 mm DV), temporal (−3.20 mm AP, 1.08 mm ML, 2.72 mm DV), inferior temporal (−3.21 mm AP, 1.08 mm ML, 2.64 mm DV) and superior (−3.19 mm AP, 1.05 mm ML, 2.82 mm DV) retina, respectively. Taken together, retrogradely labeled cPAG-projecting RGCs were distributed throughout the whole retina. The present results show that there is a density distribution variation within the retina; for instance, 58 cells/mm^2^ was seen at eccentricity 1.26 mm, 66 cells/mm^2^ at 3.47 mm and 50 cells/mm^2^ at 4.82 mm. However, the difference is not statistically significant (p = 0.83). Based on the area of dendritic field and local cell density, we calculated the coverage factor (dendritic field area × cell density) of cPAG-projecting RGCs, and obtained a figure of 3.2. As shown in Table 1, the number of cPAG-projecting RGCs required to tile up the entire retina is approximately 1300.

**Table 1:** Number of caudal periaqueductal gray (cPAG)-projecting retinal ganglion cells (RGCs) required to tile up the retinal surface.

Number of cPAG-projecting RGCs needed to tile up the retinal surface	
Average dendritic field diameter (mm)	0.248±0.038
Average dendritic field area (mm^2^)	0.048
Number of cPAG-projecting RGCs	134
Retinal area (mm^2^) [11]	64
Number of cells needed to cover the entire retinal surface	**1333**

The calculations are used to determine the number of cPAG-projecting RGCs that are required to cover the whole retinal surface. The size of the retinal surface area is referred from Fite’s study [11]; tiling up the whole retinal surface is generally considered as a criterion for a type of RGC, and our results indicate approximately 1300 cPAG-projecting RGCs are required to cover the whole retinal surface in this species.

## Discussion

Through retrograde labeling and intracellular injection techniques, the present study has characterized morphological properties of retinal ganglion cells (RGCs) projecting to the caudal periaqueductal gray (cPAG). The cPAG-projecting RGCs show unique dendritic branching and ramification patterns. For the first time we provide morphological evidence that cPAG, a multifunctional non-visual center, could receive direct retinal input from two types of bistratified RGCs in Mongolian gerbils.

### Possible homologues in other mammalian retinas

The morphological characteristics of bistratified cPAG-projecting RGCs resemble G12 cells in mice and bistratified RG_c_ “others” cells in rats, which also possess small somata and medium sized fields of dendrites with curly branches [26], [27]. G6 cells/uniformity detectors in rabbits and suppressed-by-contrast cells in cats and primates are medium-size neurons, and their dendritic arbors are irregular [13], [18], [28]–[32]. They seem to resemble our bistratified cPAG-projecting RGCs. However, cPAG-projecting RGCs lack the recurrent dendrites diving from the OFF sublamina to the ON sublamina [33]. One of the morphological characteristics of the bistratified cPAG-projecting RGCs is that the processes stratified in sublamina ***a*** are thin whereas those ramified in sublamina ***b*** are thick. We are not aware of any similar morphological characteristics of bistratified RGCs that have been reported.

There is evidence that melanopsin-expressing RGCs (mRGCs) could innervate rostral PAG (rPAG) [12], and it is well demonstrated that mRGCs can play a pivotal role in processing non-visual information [34]–[36]. One might expect that cPAG receives direct innervations from mRGCs. Although this study confirms that a small subset of mRGCs (M1) could be retrogradely labeled by CTB deposited at rPAG, our results indicate that cPAG does not receive direct innervations from mRGCs.

### Distribution pattern

**Figure 8:**
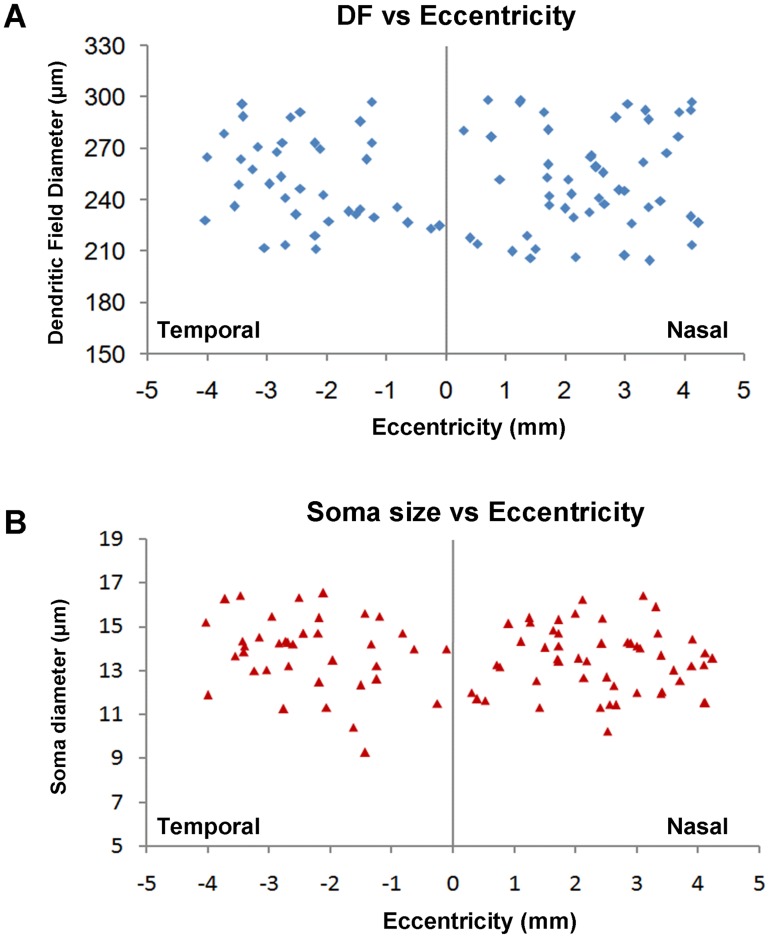
Variation in the sizes of dendritic fields and somata with retinal eccentricity. (A) Plot of dendritic field diameter as a function of eccentricity. (B) Plot of soma diameter as a function of eccentricity. 7 animals were used in each analysis.

As shown in Fig. 8, sizes of both somata and dendritic fields of cPAG-projecting RGCs do not vary with eccentricity. Due to technical difficulties in direct intracellular injection of tracer in prior retrogradely labeled RGCs within 1 mm at temporal side from the optic disk, we were unable to inject as many encountered RGCs in this region as possible. However, it should not affect the interpretation of the data because we were able to inject all encountered cells in the nasal part of the retina (1 mm from optic disk). As shown in Fig. 5F, cPAG-projecting RGCs are evenly distributed throughout the whole retina. Since the data were collected from five retinas, one may expect that the density obtained could have been underestimated. This could be the case. However, as comparing the local densities of one most peripheral area in Fig. 5F (magenta arrow) with two central areas (blue arrow and cell D), all three local areas show similar density. Thus, the present study offers a relatively accurate estimation of density of the cPAG-projecting RGCs (Table 1).

### Dendritic morphology and functional consideration

The dendritic field of cPAG-projecting RGCs was covered by loosely distributed and occasionally overlapped processes. The sizes of dendritic fields and diameters of somata of the cPAG-projecting RGCs were smaller than those of dorsal raphe nuclei (DRN)-projecting alpha cells in this species [4]. Apparent overlapping dendritic fields were found among close cPAG-projecting RGCs. These cells might be able to sample the visual field with the appropriate coverage.

Based on the classical stratification rules of inner plexiform layer (IPL), cPAG-projecting RGCs had morphological features of bistratified RGCs. This suggests that cPAG-projecting RGCs are capable of detecting visual information from both ON and OFF retinal channels. Recent developments in connectomics technologies has enriched the knowledge of retinal networks, and a “refactored IPL model” has been proposed, which contains border ON-OFF band spans 75% of the IPL [37]–[39]. It will be interesting to find out the relationship between the physiological features of cPAG-projecting RGCs and their accurate pattern of connections in the retina.

Substantial behavioral evidence has indicated that PAG neurons could regulate fear-related behavior [40]–[45]. Furthermore, Warthen and colleagues demonstrated that light could enhance learned fear in the mice, a phenomenon that was driven primarily by rods/cones [2]. However, the mechanism underlying action of light in the regulation of fear-related behavior remains to be determined. The discovery of these cPAG-projecting bistratified RGCs proposes a possibility that fear-related contrast changes in the visual field can be effectively transmitted to PAG through cPAG-projecting RGCs in Mongolian gerbils.
